# The incidences of postoperative hypoparathyroidism during thyroid surgery with ipsilateral central lymph node dissection for papillary thyroid carcinoma were close to that during thyroid surgery for benign thyroid diseases necessitating surgical intervention: a retrospective study

**DOI:** 10.3389/fendo.2024.1461553

**Published:** 2024-12-23

**Authors:** Bin Wang, Chun-Rong Zhu, Yuan Fei, Qiyue ShanZhou, Hong Liu, Xin-Min Yao, Jian Wu

**Affiliations:** ^1^ Center of Breast and Thyroid Surgery, Department of General Surgery, The Third People’s Hospital of Chengdu, Chengdu, Sichuan, China; ^2^ Department of Oncology Ward 2, The Third People’s Hospital of Chengdu, Chengdu, Sichuan, China; ^3^ Department of Hematology and Oncology, Chongzhou People’s Hospital, Chongzhou, Sichuan, China

**Keywords:** hypoparathyroidism, papillary thyroid carcinoma, thyroidectomy, prophylactic, central lymph node dissection

## Abstract

**Objective:**

This study aimed to assess the degree of effect of central lymph node dissection on postoperative hypoparathyroidism incidence.

**Methods:**

The incidence of postoperative hypoparathyroidism was compared between patients receiving thyroidectomy with central neck dissection for papillary thyroid carcinoma and those undergoing thyroidectomy for benign thyroid diseases (thyroid follicular adenoma and/or nodular goiter) necessitating surgical intervention.

**Results:**

The incidence of postoperative hypoparathyroidism was not significantly different between the groups of lobe thyroidectomy for benign thyroid diseases and lobe thyroidectomy with ipsilateral central lymph node dissection for papillary thyroid carcinoma (immediate: 9.2% vs 3.0%, P = 0.157; protracted: 4.6% vs 0%, P = 0.06; permanent: 0% vs 0%). Similarly, there was no significant difference in the postoperative hypoparathyroidism incidence between total thyroidectomy for benign thyroid diseases and total thyroidectomy with ipsilateral central lymph node dissection for papillary thyroid carcinoma (immediate: 25.0% vs 33.8%, P = 0.12; protracted: 4.5% vs 5.3%, P = 0.99; permanent: 1.1% vs 0.9%, P > 0.99).

**Conclusion:**

While the rates of postoperative hypoparathyroidism during thyroid surgery with ipsilateral central lymph node dissection may be slightly elevated compared to surgery without it for papillary thyroid carcinoma, they remained akin to those observed during surgery for benign thyroid diseases that need surgical management.

## Introduction

According to the 2015 American Thyroid Association Management Guidelines for Adult Patients with Thyroid Nodules and Differentiated Thyroid Cancer (2015 ATA Guidelines), prophylactic central neck dissection (pCND) is not recommended for small (T1 or T2), noninvasive, clinically node-negative papillary thyroid carcinoma (cN0 PTC) and for most follicular cancers (1). One rationale behind this decision is the increased likelihood of temporary morbidity associated with pCND (1, 2). However, due to the notably incidence of occult central lymph node metastasis (43.5% – 64.7%) (3–5), and the higher complications during reoperation compared to initial thyroid surgery (6, 7), along with the negative impact of recurrence on quality of life and mental well-being, ipsilateral central neck dissection (ICND) is strongly advocated for routine implementation in PTC cases where the parathyroid gland (PG) and recurrent laryngeal nerve can be effectively safeguarded, as per the Chinese Guidelines for the Diagnosis and Management of Thyroid Nodules and Differentiated Thyroid Cancer (Second edition) (8).

In consideration of the contrary recommendation between aforementioned Guidelines, a review was conducted on studies supporting the increased temporary morbidity in the 2015 ATA Guidelines (2, 9–13). Postoperative hypoparathyroidism incidence, which increased by 10%~21%, contributed the most significant increase in temporary morbidity (2, 9–13). Most patients experiencing temporary hypoparathyroidism recovered within a month through appropriate therapy, suggesting that the effect of temporary hypoparathyroidism was not severe (14). What’s more, it is the chronic/permanent hypoparathyroidism that warrants attention as highlighted in the Guidelines for the Evaluation and Management of Hypoparathyroidism (15, 16), given its association with increased risks of several diseases (17–19). Hence, the present study sought to evaluate the influence of central lymph node dissection (CND) on postoperative hypoparathyroidism during thyroid surgery for PTC. We conducted this study to compare postoperative hypoparathyroidism incidence between thyroidectomy with CND for PTC and thyroidectomy for benign thyroid diseases (thyroid follicular adenoma and/or nodular goiter) necessitating surgical intervention.

## Patients and methods

### Patients

We meticulously reviewed the records of patients who underwent thyroid surgery at our institution between July 2018 and December 2022. This retrospective study exclusively included patients who met all the following criteria: 1) initial thyroid surgery; 2) thyroid surgery for PTC, thyroid follicular adenoma, or nodular goiter; and 3) the largest diameter of the PTC ≤ 4 cm. The exclusion criteria were as follows: 1) individuals who had received neck radiation therapy; 2) those with a history of neck surgery; 3) patients with concurrent parathyroid disorders or abnormal preoperative serum parathyroid hormone (PTH) levels; and 4) patients with incomplete medical records or postoperative follow-up spanning less than 6 months. This study was approved by the Medical Ethics Committee of The Third People’s Hospital of Chengdu. Informed consent was routinely obtained from all the participants for utilizing clinical data for medical research before they were discharged.

### Surgical indications and procedures

Lobe thyroidectomy (LT) was performed for confirmed thyroid follicular neoplasms based on preoperative fine-needle aspiration biopsy and intraoperative frozen pathology. Total thyroidectomy (TT) was performed for thyroid follicular neoplasms with thyroid nodules in the contralateral lobe as indicated by preoperative image. TT was performed when the diameter of the nodular goiter was > 4cm and the diameter of the thyroid nodule in the contralateral lobe was > 2cm. Otherwise, LT was performed.

The surgical strategies for unilateral cT1~2N0 PTC have been described previously (5). Bilateral or isthmic PTC cases warranted TT with bilateral central neck dissection (BCND). TT with ICND was routinely performed for T3b~4 PTC and cN1a PTC, and contralateral central neck dissection was performed upon confirmation of capsular invasion and/or prelaryngeal and/or pretracheal lymph node metastasis through intraoperative frozen pathology. TT, BCND, and ipsilateral lateral lymph node dissection (compartments II–V) were performed for cN1b PTC.

The surgical procedures for thyroidectomy and CND were the same as those described previously (5). Lateral lymph node dissection was then performed by the intervals between the sternomastoid muscle and strap muscles, and intermuscular space of the sternomastoid muscle following CND.

### Perioperative management

Standardized perioperative management and postoperative calcium supplementation strategies have been described previously (14). Hypoparathyroidism was considered when the serum PTH level was less than the normal range (1.6–6.9 pmol/L) or when symptoms required calcium supplementation, regardless of the PTH values. Immediate hypoparathyroidism was defined as hypoparathyroidism on postoperative 1 day. When hypoparathyroidism lasted for > 1 and 6 months, it was deemed protracted hypoparathyroidism and permanent hypoparathyroidism, respectively.

### Data collection

The following data were collected: demographic characteristics, comorbidities, preoperative assessment, details of surgical extent, number of autoplastic and/or inadvertently resected PG, postoperative pathological reports, lesion characteristics, postoperative complications, and preoperative and postoperative PTH levels.

### Statistical analysis

The SPSS version 23.0 software (SPSS Inc, Chicago, IL, USA) was used for all statistical analyses. Continuous and categorical data are expressed as mean ± standard deviation (SD) and absolute numbers, respectively. Student t-test or Mann–Whitney U test was used for continuous variables, and Pearson chi-square test or Fisher exact test was used for categorical variables. Multivariate binary logistic regression analysis was used for risk factor analyses of PG autotransplantation, inadvertent resection of PG, and postoperative hypoparathyroidism. Statistical significance was set at P < 0.05.

## Results

A total of 1232 patients were included in this retrospective study, among which 65 patients underwent LT; 100, LT with ICND; 88, TT; 320, TT with ICND; 501, TT with BCND; 135, TT with BCND and unilateral neck lymph node dissection (compartments II–V) (ULND); and, 23, TT with BCND and bilateral neck lymph node dissection (compartments II–V) (BLND).

The data of patients who underwent LT was compared with that of patients who underwent LT with ICND (**Table 1**). The LT group exhibited a higher proportion of patients aged ≥55 years and with hypertension compared to the LT + ICND group (30.7% vs 9%, P < 0.001; 18.5% vs 7%, P =0.024; **Table 1**). The LT + ICND group showed higher incidences of Hashimoto’s Thyroiditis and PG autotransplantation (10.8% vs 26%, P = 0.017; 30.8% vs 47%, P = 0.038; **Table 1**). Preoperative and postoperative PTH levels were both higher in the LT cohort than the LT + ICND group, with no significant differences in decreased PTH levels post-surgery and postoperative hypoparathyroidism rates between the groups. The proportions of diabetes, hypothyroidism, or other postoperative complications (transient hoarseness, pneumonia, urinary system infection, and chylous fistula) did not differ significantly between the two groups. The postoperative hospital stay was longer in the LT group (3.9 ± 1.6 days vs 3.3 ± 1.3 days, P = 0.008; **Table 1**).

**Table 1:** The characteristics of the group of LT and LT with ICND.

Variables	LT (n=65)	LT + ICND (n=100)	P
Sex (F/M)	37/28	67/33	0.19
Age (years old)	46.5 ± 15.7	42.6 ± 10.8	0.080
≤55	45	91	<0.001
>55	20	9	
Body Mass Index	23.3 ± 3.1	23.5 ± 3.7	0.653
Hypertension	12	7	0.024
Diabetes	4	4	0.713
Hyperthyroidism	9	1	0.001
Hypothyroidism	1	2	>0.99
Hashimoto’s Thyroiditis	7	26	0.017
Preoperative PTH (pmol/l)	6.54 ± 2.93	5.59 ± 2.08	0.020
Largest size of the main lesion (mm)	51.2 ± 18.2	6.8 ± 1.9	<0.001
Autoplastic PG	0.3 ± 0.5	0.5 ± 0.5	0.045
0	45	53	0.038
≥1	20	47	
Inadvertent resection of PG	0.1 ± 0.2	0 ± 0.2	0.532
0	61	96	0.713
≥1	4	4	
Transient hoarseness	3	4	>0.99
Permanent hoarseness	0	0	—
Pneumonia	1	0	0.394
Urinary system infection	1	0	0.394
Chylous fistula	0	1	>0.99
Postoperative PTH (pmol/l)			
1 Day	4.14 ± 2.55	3.43 ± 1.21	0.038
1 Month	5.42 ± 3.11	4.28 ± 1.71	0.008
6 Months	5.52 ± 1.36	4.62 ± 1.4	<0.001
Pre-PTH minus Post-1-Day PTH (pmol/l)	2.19 ± 3.34	2.05 ± 1.67	0.755
Pre-PTH minus Post-1-month PTH (pmol/l)	0.91 ± 3.69	1.20 ± 1.96	0.562
Pre-PTH minus Post-6-months PTH (pmol/l)	0.81 ± 2.82	0.86 ± 2.36	0.906
Immediate hypoparathyroidism	6	3	0.157
Protracted hypoparathyroidism	3	0	0.059
Permanent hypoparathyroidism	0	0	—
Postoperative hospital stay (days)	3.9 ± 1.6	3.3 ± 1.3	0.008

LT, lobe thyroidectomy; ICND, ipsilateral central lymph node dissection; F, female;, M, male; PTH, parathyroid hormone; PG, parathyroid gland.

The multivariate analysis suggested that ICND was an independent risk factor for PG autotransplantation (OR = 1.995, 95% CI 1.034–3.849, P = 0.039; G =218.511, P < 0.001). However, no risk factors were found for inadvertent resection of PG (G = 61.496, P < 0.001) or postoperative immediate hypoparathyroidism (G = 66.225, P < 0.001) in the multivariate analysis of data of the LT and LT + ICND groups.

**Table 2** shows data for patients undergoing TT as the minimal surgical extent. In the TT group, the patients were older with a higher prevalence of hypothyroidism. Compared to patients who underwent only TT, there was a higher risk of PG autotransplantation in patients who underwent TT with CND (TT: 33.0%, TT + ICND: 53.8%, TT + BCND: 67.1%; P _TT vs ICND_ = 0.001, P _TT vs BCND_ < 0.001; **Table 2**) and a higher risk of inadvertent resection of PG in patients who underwent TT + BCND (TT: 3.4%, TT + BCND: 12.3%; P _TT vs BCND_ = 0.013; **Table 2**). The postoperative-1-day PTH level was higher in the TT group than those in the TT + ICND and TT + BCND groups, whereas the incidence of immediate hypoparathyroidism was not significantly different between the TT and TT + ICND groups (25.0% vs 33.8%, P = 0.12; **Table 2**). While the postoperative-1-month PTH level was higher in the TT group than that in the TT + BCND group, the incidence of protracted hypoparathyroidism did not significantly differ among the three groups. No significant differences in the incidence of permanent hypoparathyroidism were observed.

**Table 2:** The characteristics of the group of TT, TT with ICND, and TT with BCND.

Variables	TT n=88	TT + ICND n=320	TT + BCND n=659	P		
				TT vs ICND	TT vs BCND	ICND vs BCND
Sex (F/M)	74/14	234/86	469/190	0.034	0.011	0.523
Age (years old)	52.1 ± 13.6	41.5 ± 12.6	47.3 ± 13	0.003	<0.001	<0.001
≤55	51	240	575	0.002	<0.001	<0.001
>55	37	80	84			
Body Mass Index	24.3 ± 3.6	23.6 ± 3.4	23.2 ± 3.4	0.293	0.206	0.583
Hypertension	11	45	73	0.706	0.692	0.178
Diabetes	5	10	20	0.333	0.326	0.939
Hyperthyroidism	2	4	12	0.614	0.676	0.509
Hypothyroidism	16	8	24	<0.001	<0.001	0.346
Hashimoto’s Thyroiditis	17	81	204	0.244	0.025	0.068
Nodular goiter	—	195	368	—	—	0.13
Preoperative PTH (pmol/l)	5.73 ± 3.3	5.57 ± 2.9	5.59 ± 2.11	0.468	0.682	0.814
Largest size of the main lesion (mm)	41.7 ± 24.8	11.5 ± 7.8	15.9 ± 10.4	<0.001	<0.001	<0.001
Preoperative lymphadenectasis in the central zone	—	39	211	—	—	<0.001
Largest size of lymph nodes (mm)	—	11.3 ± 5	12.6 ± 5.3	—	—	0.154
Autoplastic PG	0.4 ± 0.6	0.6 ± 0.6	0.9 ± 0.8	0.003	<0.001	<0.001
0	59	148	217	0.001	<0.001	<0.001
≥1	29	172	442			
Inadvertent resection of PG	0 ± 0.2	0.1 ± 0.3	0.1 ± 0.3	0.048	<0.001	0.041
0	85	294	578	0.127	0.013	0.050
≥1	3	26	81			
Transient hoarseness	0	12	14	0.078	0.336	0.138
Permanent hoarseness	0	3	0	>0.99	—	0.035
Wound infection	2	1	4	0.119	0.313	>0.99
Pneumonia	2	1	9	0.119	0.847	0.231
Urinary system infection	0	3	5	>0.99	>0.99	0.721
Postoperative bleeding	1	2	6	0.519	0.586	0.931
Chylous fistula	0	0	4	—	>0.99	0.31
Postoperative PTH (pmol/l)						
1 Day	2.71 ± 1.59	2.35 ± 1.43	1.9 ± 1.25	0.040	<0.001	<0.001
1 Month	4.23 ± 1.64	3.91 ± 1.63	3.71 ± 1.56	0.099	0.004	0.074
6 Months	4.33 ± 1.73	4.26 ± 1.69	4.4 ± 1.76	0.732	0.724	0.236
Immediate hypoparathyroidism	22	108	321	0.119	<0.001	<0.001
Protracted hypoparathyroidism	4	17	36	0.987	0.915	0.922
Permanent hypoparathyroidism	1	3	8	>0.99	>0.99	0.951
Postoperative Hospital stay (days)	4.1 ± 2.5	3.8 ± 1.8	4.9 ± 3.5	0.345	0.052	<0.001

TT, total thyroidectomy; ICND, ipsilateral central lymph node dissection; BCND, bilateral central lymph node dissection; F, female; M, male; PTH, parathyroid hormone; PG, parathyroid gland.

Compared with patients in the TT + ICND group, patients in the TT + BCND group exhibited a higher the incidence of PG autotransplantation and immediate hypoparathyroidism (53.8% vs 67.1%, P < 0.001; 33.8% vs 48.7%, P < 0.001; **Table 2**). However, there was no significant difference in the incidence of inadvertent resection of PG between the TT + ICND and TT + BCND groups. The postoperative-1-day PTH level was lower in the TT + BCND group; however, the postoperative-1-month and postoperative-6-month PTH level did not differ significantly between the two groups. The incidence of preoperative lymphadenectasis in the central zone (12.2% vs 32.0%, P < 0.001) and postoperative hospital stay (3.8 ± 1.8 vs 4.9 ± 3.5, P < 0.001) differed significantly between the two groups.

Analyses of the risk factors for postoperative immediate hypoparathyroidism, PG autotransplantation, and inadvertent resection of PG from patients who underwent TT with or without ICND were shown in **Table 3**. The multivariate analysis demonstrated that female sex (OR = 2.64; 95%CI 1.49 – 4.67; p = 0.001), lower age (> 55 years old, OR = 0.38; 95%CI 0.22 – 0.64; p < 0.001), and inadvertent resection of PG (OR = 2.44; 95% CI 1.08 – 5.51; p = 0.033) were independent risk factors for postoperative immediate hypoparathyroidism (**Table 3**). ICND was confirmed to be a risk factor for PG autotransplantation (OR = 2.36; 95% CI 1.44 – 3.88; p = 0.001; **Table 3**) and inadvertently removed PG (OR = 7.69; 95% CI 1.03 – 57.51; p = 0.047; **Table 3**).

**Table 3:** Multivariate analysis of risk factors for postoperative immediate hypoparathyroidism, PG autotransplantation, and inadvertent resection of PG from data of the group of TT and TT with ICND.

Variables	Postoperative immediate hypoparathyroidism (G = 477.493, P<0.001)			PG autotransplantation (G=553.371, P < 0.001)			Inadvertent resection of PG (G=191.303, P < 0.001)		
	OR	95%CI	P	OR	95%CI	P	OR	95%CI	P
ICND				2.36	1.44 - 3.88	0.001	7.69	1.03 - 57.51	0.047
Female	2.64	1.49 - 4.67	0.001						
Age (> 55 years old)	0.38	0.22 - 0.64	<0.001						
Inadvertent resection of PG	2.44	1.08 - 5.51	0.033						

TT, total thyroidectomy; ICND, ipsilateral central lymph node dissection; PG, parathyroid gland.

BCND (OR = 1.46; 95% CI 1.12 – 1.9; p = 0.005), female sex (OR = 1.47; 95%CI 1.05 – 2.06; p = 0.027), PG autotransplantation (OR = 2.53; 95% CI 1.83 – 3.49; p < 0.001), and inadvertent resection of PG (OR = 1.98; 95% CI 1.21 – 3.23; p = 0.006) were independent risk factors for postoperative immediate hypoparathyroidism according to the multivariate analysis of the data of patients who underwent TT with or without BCND (including ULND and BLND). BCND was a risk factor for both PG autotransplantation (OR = 2.04; 95% CI 1.61 – 2.58; p < 0.001; **Table 4**) and inadvertently removed PG (OR = 3.49; 95% CI 1.29 – 9.42; p = 0.014; **Table 4**).

**Table 4:** Multivariate analysis of risk factors for postoperative immediate hypoparathyroidism, PG autotransplantation, and inadvertent resection of PG from data of the group of TT and TT with BCND.

Variables	Postoperative immediate hypoparathyroidism (G = 967.262, P<0.001)			PG autotransplantation (G=942.418, P < 0.001)			Inadvertent resection of PG (G=502.149, P < 0.001)		
	OR	95%CI	P	OR	95%CI	P	OR	95%CI	P
BCND	1.46	1.12 - 1.9	0.005	2.04	1.61 - 2.58	<0.001	3.49	1.29 - 9.42	0.014
Female	1.47	1.05 - 2.06	0.027						
PG autotransplantation	2.53	1.83 - 3.49	<0.001						
Inadvertent resection of PG	1.98	1.21 - 3.23	0.006						

TT, total thyroidectomy; BCND, bilateral central lymph node dissection; PG, parathyroid gland.

When the multivariate analyses were performed among patients who underwent TT with ICND and TT with BCND (including ULND and BLND), BCND (OR = 1.59; 95% CI 1.19 – 2.12; p = 0.002; **Table 5**), female sex (OR = 1.72; 95%CI 1.27 – 2.32; p < 0.001; **Table 5**), lower age (> 55 years old, OR = 0.51; 95%CI 0.35 – 0.75; p = 0.001; **Table 5**), PG autotransplantation (OR = 2.31; 95% CI 1.74 – 3.06; p < 0.001; **Table 5**), and inadvertent resection of PG (OR = 2.2; 95% CI 1.43 – 3.38; p < 0.001; **Table 5**) were independent risk factors for postoperative immediate hypoparathyroidism. BCND (OR = 1.59; 95%CI 1.2 – 2.11; p = 0.001; **Table 5**) and preoperative lymphadenectasis in the central zone (OR = 1.79; 95%CI 1.29 – 2.49; p < 0.001; **Table 5**) were both highlighted as risk factors for PG autotransplantation. No risk factors for inadvertent resection of PG were identified (G = 668.926, P < 0.001).

**Table 5:** Multivariate analysis of risk factors for postoperative immediate hypoparathyroidism, PG autotransplantation, and inadvertent resection of PG from data of the group of TT with ICND and TT with BCND.

Variables	Postoperative immediate hypoparathyroidism (G =1249.409, P<0.001)			PG autotransplantation (G=1259.575, P < 0.001)			Inadvertent resection of PG (G=668.926, P < 0.001)		
	OR	95%CI	P	OR	95%CI	P	OR	95%CI	P
BCND	1.59	1.19 - 2.12	0.002	1.59	1.2 - 2.11	0.001			
Female	1.72	1.27 - 2.32	<0.001						
Age (> 55 years old)	0.51	0.35 - 0.75	0.001						
Preoperative lymphadenectasis in the central zone				1.79	1.29 - 2.49	<0.001			
PG autotransplantation	2.31	1.74 - 3.06	<0.001						
Inadvertent resection of PG	2.20	1.43 - 3.38	<0.001						

TT, total thyroidectomy; ICND, ipsilateral central lymph node dissection; BCND, bilateral central lymph node dissection; PG, parathyroid gland.

## Discussion

The direct outcome of PG injury was the inability to preserve the PG at its original site (including PG autotransplantation and inadvertent resection of the PG). The overall outcome was postoperative hypoparathyroidism. The present study suggested that CND was a risk factor for PG autotransplantation and increased the risk of inadvertent resection of PG in patients who underwent TT as the minimum extent of surgery. Female sex and inadvertent PG removal were established as risk factors for postoperative immediate hypoparathyroidism after TT with or without CND. The influence of PG autotransplantation in postoperative immediate hypoparathyroidism became significant after BCND.

In comparison to thyroidectomy, thyroidectomy with CND involves a larger surgical extent, potentially heightening the risk of PG injury during PTC surgery (10, 13, 20, 21). In this study, the lesion size of benign thyroid diseases was larger than that of PTC, indicative of larger surgical area during thyroidectomy. However, when CND was performed, the number and incidence of autoplastic PG were higher in the PTC group. This might be because CND increased the risk of injury to PG blood supply, and the increased risk was higher than that from the larger lesion size (21). The degree of difficulty in preserving PG *in situ* differs among different types of PG (22).

The present study suggests that the effect of CND on inadvertent resection of PG was significant among patients undergoing TT with or without CND, which is consistent with previous studies on PTC (23–25). More tissue was removed in CND. Although the influence of surgery on larger lesions in the surrounding tissue is greater, resection of the surrounding lymphatic and adipose tissues is not required for benign thyroid diseases. Therefore, the increased risk of CND for inadvertent resection of PG surpassed that associated with larger benign lesion sizes. However, the effect of ICND on inadvertent resection of PG was not significant in patients undergoing LT with or without ICND, possibly due to the small sample size in this subgroup.

The effect of ICND on postoperative immediate hypoparathyroidism was not confirmed to be significant when it was added to LT or TT in this study, whereas the effect of BCND and an additional lateral CND was confirmed to be significant when it was added to TT and TT + UCND, respectively. The postoperative-1-day PTH level was decreasing with the additional lateral CND. This phenomenon might be explained by the fact that the effect of ICND did not exceed the reserve capacity of PGs in the LT + ICND and TT + ICND groups when compared with that of LT and TT, whereas the effect of BCND and an additional lateral CND exceeded the reserve capacity of PGs in the TT+BCND group when compared with TT and TT + ICND groups.

The inadvertent resection of PG increased the risk of postoperative immediate hypoparathyroidism in patients undergoing TT with or without CND. In general, one patient had 4 PGs. The inadvertent resection of PG decreased the number of functional PGs, which would increase the risk of postoperative hypoparathyroidism, especially in those undergoing TT with or without CND, which might cause damage to all the PG. Previous studies have concluded based on the data of surgery for PTC (23–25). The present study suggests that the risk of postoperative immediate hypoparathyroidism caused by a larger lesion size could not cover the risk by inadvertent resection of PG. However, in patients who underwent LT with or without ICND, inadvertent resection of PG did not significantly affect postoperative immediate hypoparathyroidism. This effect might be due to the unaffected contralateral PGs in surgery.

Several studies have suggested that female sex is a risk factor for postoperative immediate hypoparathyroidism (14, 26–28). Similar result was obtained in the present study. The differences in the constituent ratio of tissues, weight, and factors influencing the secretion of PG between women and men were speculated to be the reasons for this (14, 27–29). With the inclusion of patients who underwent TT + BCND in our analysis, PG autotransplantation emerged as a notable risk factor for immediate postoperative hypoparathyroidism. Almost all PGs and/or their blood supply were affected by TT+BCND; therefore, the function of the autoplastic PG could not be compensated for in a timely manner by the other PGs and then the influence of PG autotransplantation became significant. The association between age and immediate postoperative hypoparathyroidism could be attributed to incidental factors, a relationship not previously delineated in studies. In the presence of preoperative lymphadenectasis in the central zone, the surgeon might perform more radical CND, thereby heightening the likelihood of PG autotransplantation.

## Conclusion

In conclusion, compared with that in thyroid surgery without ICND for PTC, the incidence of postoperative immediate hypoparathyroidism might be elevated in thyroid surgery with ICND for PTC, but it remains comparable to that in thyroid surgery for benign thyroid diseases necessitating surgical intervention. This indicated that the increased risk of postoperative immediate hypoparathyroidism from ICND was deemed acceptable and ICND was a safe surgery that could be performed to allay concerns regarding reoperation and recurrence. When compared to thyroidectomy for benign thyroid diseases necessitating surgical intervention, thyroidectomy with CND for PTC increased the risk of PG autotransplantation and inadvertent PG resection. Female sex, inadvertent PG resection, and PG autotransplantation increased the risk of postoperative immediate hypoparathyroidism.

## Data Availability

The raw data supporting the conclusions of this article will be made available by the authors, without undue reservation.
